# Epidemiology and outcomes from severe hypoglycemia in Kuwait: a prospective cohort study

**DOI:** 10.1186/s12873-021-00457-9

**Published:** 2021-05-29

**Authors:** Dalal Al Hasan, Ameen Yaseen, Mohammad Al Roudan, Lee Wallis

**Affiliations:** 1 Department of Applied Medical Sciences, College of Health Sciences, Public Authority of Applied Education and training, State of Kuwait, Kuwait City, Kuwait; 2 Audit Department, Emergency Medicals Services, State of Kuwait, Kuwait City, Kuwait; 3 Emergency Medicine Department, University of Cape Town, Cape Town, South Africa

**Keywords:** Severe hypoglycemia, Emergency medical services, Glasgow coma scale, Random blood sugar, Kuwait

## Abstract

**Background:**

The objective of this study was to describe the epidemiology of severe hypoglycaemia in Kuwait, aiming to provide a preliminary background to update the current guidelines and improve patient management.

**Method:**

This was a prospective analysis of severe hypoglycaemia cases retrieved from emergency medical services (EMS) archived data between 1 January and 30 June 2020. The severe hypoglycaemia cases were then sub-grouped based on EMS personal initial management and compared in terms of scene time, transportation rate, complications and outcomes. The primary outcomes were GCS within 10–30 min and normal random blood glucose (RBS) within 10–30 min.

**Results:**

A total of 167 cases met the inclusion criteria. The incidence of severe hypoglycaemia in the national EMS was 11 per 100,000. Intramuscular glucagon was used on scene in 89% of the hypoglycaemic events. Most of the severe hypoglycaemia patients regained normal GCS on scene (76.5%). When we compared the two scene management strategies for severe hypoglycaemia cases, parenteral glucose administration prolonged the on-scene time (*P = .002)* but was associated with more favourable scene outcomes than intramuscular glucagon, with normal GCS within 10–30 min *(P = .05)* and normal RBS within 10–30 min *(P = .006)*. Conclusion: Severe hypoglycaemia is not uncommon during EMS calls. Appropriate management by EMS personals is fruitful, resulting in favourable scene outcomes and reducing the hospital transportation rate. More research should be invested in improving and structuring the prehospital management of severe hypoglycaemia. One goal is to clarify the superiority of parenteral glucose over intramuscular glucagon in the prehospital setting.

**Supplementary Information:**

The online version contains supplementary material available at 10.1186/s12873-021-00457-9.

## Introduction

Kuwait has a high diabetes mellites incidence, with one in every five adults in Kuwait diagnosed with diabetes mellites [1]. A common diabetic emergency is hypoglycaemia [2]. Sixty-five percent of diabetic patients experience at least one episode of hypoglycaemia annually [2]. The annualized crude incidence of hypoglycaemia is 35.1 events per person-year [2]. Hypoglycaemia can be a severe or non-severe event. Severe hypoglycaemia is defined as having low blood glucose levels that require assistance from another person to treat [3]. The condition can be life-threatening, accounting for 10% of all diabetes-related mortalities [4].

In the United States, hypoglycaemic events requesting emergency medical services (EMS) place a significant burden on medical resources. Approximately 5% of all EMS calls are for hypoglycaemia [5]. EMS personnel are the first health professionals contacting the hypoglycaemic event. Their appropriate management of hypoglycaemia prevents life-threatening complications and eliminates unnecessary transports to emergency departments [6].

To the best of our knowledge, there have been a limited number of studies on severe hypoglycaemia in the prehospital setting [7, 8]. The goal of this study is to establish severe hypoglycaemia characteristics, complications and outcomes with the aim to deliver groundwork to revise the current severe hypoglycaemia guidelines.

## Methods

### Setting

Kuwait has a centralized dispatch centre for all ambulance services. For emergency calls, the universal emergency number (1–1-2) has automatic location identification with a centralized dispatch for police, fire and emergency medical services (EMS). If medical assistance is required, the call is forwarded to an EMS call-taker, who answers the call, reconfirms the address, gives first aid instructions and activates the nearest ambulance. The dispatched ambulance is staffed with two emergency medical technicians (EMTs) or 1 paramedic and 1 EMT. EMTs provide basic life support, and paramedics provide advanced life support, both based on North American resuscitation guidelines. Both EMTs and paramedics are trained in blood glucose measurement and interpretation. The national EMS provides a glucometer kit in all ambulances. For severe hypoglycaemia management, the Kuwait EMS follows different protocols than the NAEMSP recommendations for severe hypoglycaemia. In the Kuwait EMS, ‘For a patient confirmed to be hypoglycaemic and oral administration is contraindicated, the usage of 1 mg intramuscular glucagon by EMT and the usage of intravenous or intraosseous dextrose for paramedics are warranted to reverse severe hypoglycaemia in adults.’ [9] For transport decisions, the local EMS adheres to the National Model of EMS Clinical Guidelines: ‘the patient should not be transported if hypoglycaemia resolves after treatment. Release after treatment is indicated when all of the following conditions are met: random blood sugar (RBS) greater than 4.4 mol/dl (>80 mg/dl), normal Glasgow coma scale (GCS), ability to tolerate oral intake, presence of social support, and no other major compliant such as chest pain, shortness of breath, or neurological deficit.’ [10]

### Design

This was a prospective analysis of severe hypoglycaemia cases retrieved from emergency medical services (EMS) archived data between 1 January and 30 June 2020. The severe hypoglycaemia cases were then sub-grouped based on EMS personal initial management and were compared in terms of scene time, transportation rate, complications and outcomes. The primary outcomes were GCS within 10–30 min and normal RBS within 10–30 min.

In this analysis we defined favourable on-scene outcomes with severe hypoglycaemia as: normal GCS after 10–30 min on scene and normal RBS on scene after 10–30 min.

### Participants

The study population included all adult (> 18 years old) patients for whom EMS was activated due to severe hypoglycaemia. We defined severe hypoglycaemia as having low blood glucose levels of 3.9 mmol/dl or less (< 70 mg/dl) that required assistance from another person to be treated. All patients complaining of loss of consciousness, altered mental status, dizziness, seizures, generalized weakness and cerebrovascular accident mimic symptoms plus low RBS (3.9 mmol/dl or less) were included in the analysis [3].

### Data collection/measurement

Patient report forms were the only data source for severe hypoglycaemia cases. Patient report forms were completed on scene by EMS personnel and then stored in EMS audit department archived files. The researcher manually collected patient report forms from the EMS audit department archived data. All data were presented in the patient report form, including patient demographics, clinical presentation, RBS, GCS, management, transportation to the hospital and complications. We defined severe hypoglycaemia complications as head injury, cardiac arrest and airway compromise.

Patient report forms with RBS equal to or less than 3.9 mmol/dl at the initial on-scene assessment and presenting compliance with loss of consciousness, altered mental status, dizziness, seizures, generalized weakness and cerebrovascular accident mimic symptoms were included in the analysis [3].

For management, all patients were analysed for receiving intramuscular glucagon and parenteral glucose administration. Intramuscular glucagon is administered at 1 mg into the vastus lateralis muscle for a diabetic patient who is hypoglycaemic and unable to tolerate oral intake [10]. Parenteral glucose is the administration of intravenous 10% dextrose solution (150 ml) to a diabetic patient who is hypoglycaemic and not able to tolerate oral intake [4].

In relation to patient transportation to the hospital, patients were categorized as transported to the hospital, refused transportation to the hospital, and left on scene. EMS personnel leave a patient on scene if the patient meets the “treat and release” criteria [7].

Recurrent utilization of EMS was reported if the same patient repeatedly activated EMS for severe hypoglycaemia.

Severe hypoglycaemia complications included cardiac arrest, head injury or airway compromise.

In terms of primary outcomes, all cases that had a GCS of 15 at 10–30 min on scene were recorded as having a normal GCS after 10–30 min. All cases that had an RBS of 4.4 mmol/dl or more at 10–30 min on scene were documented as normal RBS after 10–30 min*.*

During the project, all data were kept in password-locked computer files that only the research investigator could open. No data sharing was allowed outside the context of this project.

### Sample size

The sample size was determined using G power software version 3.1 with an effect size = .03, power = .80, and α = .05, and the calculated sample size was approximately 82. The relevant graph is attached in Appendix A. Using convenience sampling, all eligible severely hypoglycaemic patients treated by EMS during the study period were included.

### Statistical methods

Statistical analysis was performed using Excel and Statistical Package for Social Sciences (IBM SPSS Version 23, NY, USA). Descriptive statistics such as numbers (N), percentages (%), means and SDs were determined to summarize the patients’ characteristics, dural variation, time on scene, and recurrent utilization of EMS. Severe hypoglycaemia cases were then sub-grouped based on scene management strategies and compared using Fisher’s exact test for dichotomous variables and Student’s t-test for continuous variables. Two-sided tests were applied, and a *p*-value ≤0.05 was interpreted as statistically significant. Missing data were retained as missing; i.e., they were not imputed or estimated.

## Results

**Fig. 1:**
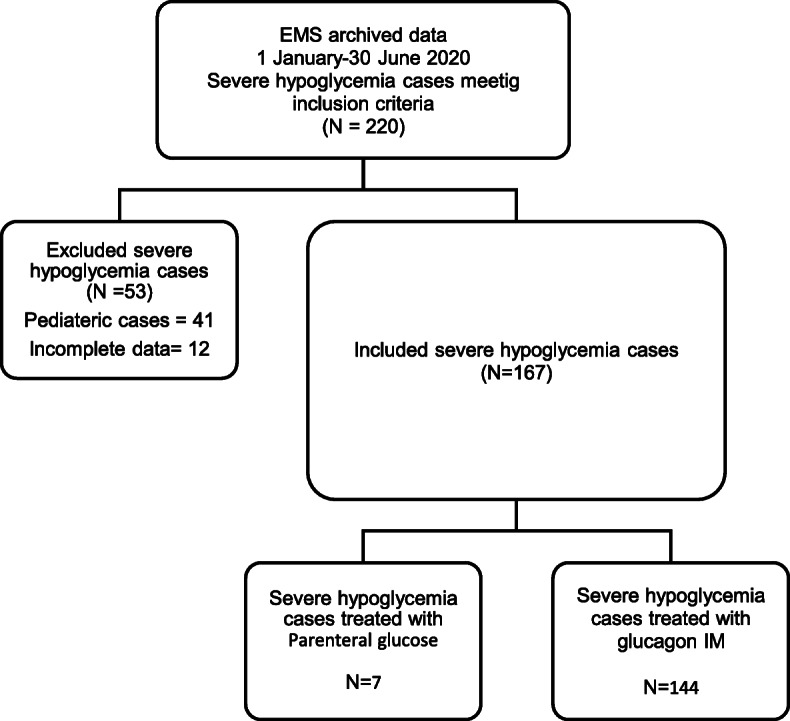
Flow chart of study population

Out of the 220 severe hypoglycaemia cases, 167 met the inclusion criteria (Fig. 1); thus, the incidence of severe hypoglycaemia was 11 per 100,000 in the national EMS. Severe hypoglycaemia patients were more likely to be Kuwaiti (63%), middle-aged (51 ± 17), and male (60.5%). The peak hours for severe hypoglycaemia events were 6:00–11:59 (27%) and 12:00–17:59 (31%) (Table 1). Intramuscular glucagon was used on scene in 89% of hypoglycaemic events. Most of the severe hypoglycaemia patients regained normal GCS on scene (76.5%, Table 2).

**Table 1:** Severe hypoglycemia patients’ demographics, clinical presentations, recurrent utilization of EMS, complications

Variables	Severe Hypoglycemia *N* = 167 (%)
Demographics	
1. Gender	
a.Male	98 (60.5)
b.Female	64 (39.5)
2.Nationality	
a.Kuwaiti	102 (63)
b.Non Kuwaiti	60 (37)
Age (mean ± std.)	51 ± 17
3. Location	
a. Home	153 (95)
b. Public	9 (5)
Random blood glucose level	
(mmol/dl) (mean ± std.)	1.1 ± .9
Diurnal variation	
a.6:00–11:59	47 (27)
b.12:00–17:59	50 (31)
c.18:00–23:59	38 (23.5)
d.00:00–06:00	27 (16.5)
Scene time (mean ± std.)	28 ± 13
Clinical presentation	
Primary compliant	
a. loss of consciousness	59 (36.4)
b. Altered mental status	80 (49.4)
c. dizziness	2 (1.2)
d. Weakness	18 (11.1)
e. Cerebrovascular accident mimic symptoms	2 (1.2)
f. seizures	1(.6)
Transportation to the hospital	
a. Transported to hospital	55 (34)
b. Patient refused transportation	85 (52.5)
. Left on scene	22 (13.5)
Recurrent utilization of EMS	27 (17)

**Table 2:** Severe Hypoglycemia management and outcomes in the prehospital setting

Variable	Severe Hypoglycemia cases *N* = 167 (%)
Management	
a. Glucagon	144 (89)
b. Parenteral glucose	7 (4)
c. Glucagon followed by parenteral glucose	11 (7)
Outcomes	
Normal GCS^a^ with in 10–30 min	124 (76.5)
Normal RBS^a^ with in 10–30 min	80 (49.5)

^a^*GCS* Glasgow coma scale, *RBS* Random blood sugar

When we compared the two on-scene management strategies for severe hypoglycaemia cases, only few participants received parenteral glucose, 4%. Incidentally we have observed the parenteral glucose administration prolonged the on-scene time (*P = .002).* However, it was associated with more favourable scene outcomes than intramuscular glucagon, with normal GCS within 10–30 min *(P = .05)* and normal RBS within 10–30 min *(P = .006)* (Table 3). There was no significant difference between the two sub-groups in terms of intervention complications and hospital transportation rate.

**Table 3:** A comparison between severe hypoglycemia cases subgroups in terms of time on scene, complications and outcomes, Using Fisher Exact test

Variable	Intramuscular glucagon group *N* = 144 (%)	Parenteral glucose group *N* = 7 (%)	*P* value (CI = 95%)
Time on scene	27 ± 13	43 ± 14	.002
Normal GCS^a^ with in 10–30 min	109 (75)	7 (100)	.05
Normal RBS^a^ with in 10–30 min	68 (47)	7 (100)	.006
Severe hypoglycemia complications	4 (2.7)	1 (14)	.214
Intervention complications	2 (1.4)	0 (0)	.09
Transportation to hospital	48 (33)	2 (28)	.96

^a^*GCS* Glasgow coma scale, *RBS* Random blood sugar

## Discussion

The study describes severe hypoglycaemia epidemiology in Kuwait, which has not been described before. Our results showed that severe hypoglycaemia resulting in a request for EMS is common in Kuwait EMS emergencies. Eleven per 100,000 EMS calls were for severe hypoglycaemia. These results are lower than those reported in the current literature, 35.2 per 100,000 inhabitants [2]. These results were not predicted, especially with Kuwait’s high diabetes mellitus incidence rates [1] and Al Hasan et al.’s 2020 population-based study results. The author documented hypoglycaemia as the most common medical emergency in Kuwaiti homes [11].

This research also reports an overall favourable on-scene outcome for severe hypoglycaemia. A total of 76.5% of the patients regained normal GCS on scene, and 49.5% of them regained normal RBS on scene. To the best of our knowledge, this is the first study to report severe hypoglycaemia on-scene outcomes.

The favourable on-scene outcomes resulted in a lower hospital transportation rate of 34%. These rates are better than those in the United States. Fifty-three percent of hypoglycaemia patients in the United States are treated and transported by EMS [6].

Our study also reports higher rates of recurrent EMS utilization for severe hypoglycaemia than recent studies, 10% [7].

We also identified intramuscular glucagon as the most frequently used agent during on-scene management to reverse severe hypoglycaemia in Kuwait. This is different from North American EMS systems, where parenteral glucose is widely used to reverse severe hypoglycaemia [6, 8]. One reason for this discrepancy is that the majority of the local EMS staff are EMTs, and parenteral glucose administration is not within the scope of EMT practice [12].

Our analysis incidentally shows more favourable on-scene outcomes with parenteral glucose in a small group of severe hypoglycaemia patients. These findings add to Kauffman 2018 et al.’s large-scale study results. Their study declared that the use of parenteral glucose was associated with lower hospital transportation rates. However, the study did not directly assess parenteral glucose on-scene outcomes. However, these results conflict with existing evidence on the intramuscular glucagon equivalency to parenteral glucose in restoring normal blood glucose and consciousness levels [13–15]. We recommend larger scale studies to confirm favourable on-scene outcomes with parenteral glucose.

The demographic characteristics of the study population (males, 60.5% mean age, 51 ± 17 years) were similar to those of other studies (males, 56.2% mean age 55 years) [7].

Our analysis illustrates similar locations for hypoglycaemic events in the present literature [8] but different peak hours: 06:00–11:59 and 12:00–17:59. These peak hours are not in line with the predicted morning elevation of blood glucose level, i.e., the “dawn phenomenon” [16, 17]. More research is required in this area.

Collectively, this cohort is the first in the current literature to report overall favourable on-scene outcomes with severe hypoglycaemia: normal GCS after 10–30 min on scene and normal RBS on scene after 10–30 min. It also contributes to identifying the role of parenteral glucose in reducing hospital transportation rates.

This research is subjected to several limitations. It compared severe hypoglycaemia on-scene outcomes between two management groups. Consequently, it did not have a randomized, controlled design. Thus, the possibility that the associations identified were related to other factors linked to both the intervention and the outcome could not be fully eliminated. Another limitation is that missing cases were excluded from the analysis, which can introduce reporting bias. An additional limitation is that although the study highlighted that parenteral glucose administration reduced the hospital transportation rate, further research is required to establish the impact of parenteral glucose on hospital transportation rates.

## Conclusion

Severe hypoglycaemia is not uncommon during EMS calls. Appropriate management by EMS personnel is fruitful, resulting in favourable scene outcomes and reducing the hospital transportation rate. More research should be invested in improving and structuring the prehospital management of severe hypoglycaemia. One goal is to clarify the superiority of parenteral glucose over intramuscular glucagon for EMS.

## Supplementary Information

**Additional file 1.**

## Data Availability

The dataset used and analysed are available form the corresponding author on a reasonable request.

## Abbreviations

EMS: Emergency medical services
GCS: Glasgow coma scale
RBS: Random blood sugar
EMT: Emergency medical technician
