# Supplementary figures and images for: Epidemiology and outcomes from severe hypoglycemia in Kuwait: a prospective cohort study
Source: BMC Emerg Med. 2021 May 29;21:65. doi: 10.1186/s12873-021-00457-9 (PMC8164757; doi:10.1186/s12873-021-00457-9)

Appendix A. study sample size calculation using Gpower Software version 3.1.


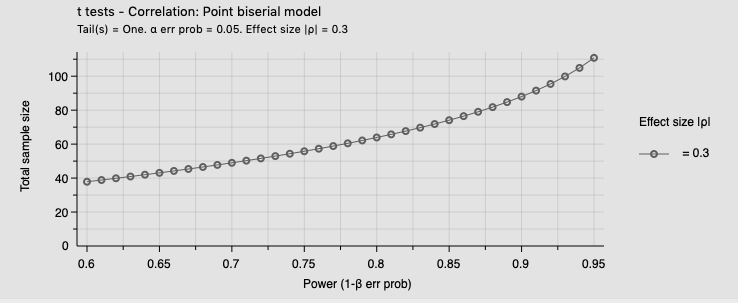

Supplement: Supplementary file 1 — Additional file 1. [file 12873_2021_457_MOESM1_ESM.docx]
